# Continuity of Care and Healthcare Costs among Patients with Chronic Disease: Evidence from Primary Care Settings in China

**DOI:** 10.5334/ijic.5994

**Published:** 2022-10-12

**Authors:** Di Liang, Wenjun Zhu, Yuling Qian, Donglan Zhang, Jindong Ding Petersen, Weijun Zhang, Jiayan Huang, Yin Dong

**Affiliations:** 1 School of Public Health, Fudan University, Key Lab of Health Technology Assessment, National Health Commission, Shanghai, China; 2 Division of Health Services Research, Department of Foundations of Medicine, NYU Long Island School of Medicine, New York, USA; 3 Research Unit for General Practice, Department of Public Health, University of Copenhagen, Copenhagen, Denmark; 4 Research Unit for General Practice, Department of Public Health, University of Southern Denmark, Denmark; 5 School of Public Health and One Health, Hainan Medical University, China; 6 David Geffen School of Medicine, University of California, Los Angeles, California, USA; 7 The People’s Hospital of Yuhuan, Zhejiang, China

**Keywords:** continuity of care, healthcare cost, primary care, China

## Abstract

**Background:**

Though critical to primary care, continuity of care has rarely been examined in China. This study aims to assess the relationship between continuity of care and healthcare costs among patients with chronic diseases within primary care settings in China.

**Methods:**

In this cross-sectional study, we used a social health insurance claims dataset of 1406 patients with hypertension and/or diabetes in Yuhuan City, Zhejiang Province collected in 2017–2019. We measured continuity of care using the Bice-Boxerman Continuity of Care (COC) Index, Herfindahl Index (HI), Sequential Continuity of Care (SECON) Index, Usual Provider of Care (UPC), and a binary variable indicating whether a patient’s UPC was a primary care provider. We examined the associations between continuity of care and healthcare costs in the same period and the subsequent year, using ordinary least squares regression for the outpatient costs and two-part regression for the inpatient costs. Based on the regression coefficients, we predicted costs saved if each continuity measure was set to 1 from the status quo.

**Results:**

When optimum continuity were to be achieved, 7.12–27.29% of total outpatient costs and 55.38–73.35% of total inpatient costs could be saved compared to the status quo during the two-year study period. If optimum continuity were to be achieved in the first year, 7.47%–21.78% of total outpatient costs and 8.84–40.22% of total inpatient costs could be saved in the second-year.

**Conclusions:**

Care continuity indicators were consistently associated with reduced outpatient costs and hospitalization risks. Future health reform in China should further enhance continuity of care in primary care.

## Background

Continuity of care is a crucial but often neglected component when we target strengthening primary care in low- and middle-income countries (LMICs) [1]. Continuity of care reflects the extent to which a series of discrete healthcare events is experienced by patients as coherent and interconnected over time and consistent with their health needs [2]. Continuity of care is a comprehensive concept, and its most important aspect is interpersonal continuity which results in trusting relationships between patients and their physicians [3]. As included in the Starfield 4C principle of primary care (first-contact access, care coordination, comprehensiveness, and continuity), continuity of care is particularly critical to the management of non-communicable diseases (NCDs) [4]. It has been widely reported that increased continuity improved the experience of care for both patients and providers, the quality of care, and health outcomes [5,6]. However, continuity of care has received relatively little attention within LMICs. Historically, health systems in LMICs primarily provided acute, episodic care, with little capacity for longitudinal follow-up and care coordination [7]. Furthermore, much of the current evidence is from managed care settings in high-income countries [8,9]. Little has been studied about continuity of care and its impact on policy-relevant outcomes (e.g., healthcare costs) in LMICs including China.

In the last decade, healthcare reform in China has focused on strengthening primary care to reshape its hospital-centric, fragmented, and inefficient delivery system [10]. Healthcare in China was primarily provided by hospitals and primary care facilities (i.e., township health centers and village clinics in rural areas and community health centers and stations in urban areas). While hospitals primarily provided acute and specialty care, primary care facilities were responsible for providing medical services as well as Basic Public Health Services including mostly preventive care [11]. In most circumstances, there were no formal gate-keeping and referral mechanisms between primary care facilities and hospitals. In 2016, a People-Centered Integrated Care (PCIC) model was introduced in a proposal regarding China’s future delivery system, a system providing the continuum of prevention, treatment, and rehabilitation services at different levels and sites of care throughout people’s life course [7,12]. To achieve these goals, a series of policies have been issued to strengthen the workforce for primary care, realign incentives in provider payment [13], and reshape the healthcare delivery system. For instance, in 2016, China’s central government issued a policy to promote “family doctor contracting services” nationally [14]. “Family doctor contracting services” – including both medical and preventive care – were primarily provided by generalist physicians along with other healthcare providers (e.g., nurses, pharmacists) for target groups such as the elderly population and patients with chronic diseases in primary care settings. Each family doctor team can contract with up to 2000 residents [15]. By the end of 2020, there were more than 430 thousand family doctor teams serving millions of people in China [16].

However, although continuity of care is a core characteristic of PCIC, continuity of care and its association with policy-relevant outcomes (e.g., healthcare costs) remained unknown in China. Few studies have measured continuity of care in China. To our knowledge, only one study examined the association between continuity of care and health-related quality of life based on self-reported data [17]. Furthermore, findings from previous literature in other countries might not be generalizable to China for the following reasons. First, even for patients with contracted family doctors, referrals are usually not required for referring patients from primary care to hospital specialists or vice versa. This is the fundamental difference in the healthcare system between China and U.S. or U.K [18]. It is unclear whether the relationship between continuity of care and healthcare costs is still held with no formal gate-keeping and referral mechanisms in the healthcare system. Second, although about 95% of people in China were covered by social health insurance programs, out-of-pocket payments still consisted of about 28.36% of national health expenditure [19]. It is also unknown to what extent continuity of care was associated with total healthcare costs, insurance-reimbursed costs, and out-of-pocket costs, respectively.

This study aimed to examine the association of continuity of care with healthcare costs among patients with chronic disease, using social health insurance claims data in Yuhuan City, Zhejiang Province, China. Yuhuan was located on the east coast of China, with 631 thousand residents and a GDP per capita of 98094 RMB in 2019 [20]. Although Yuhuan’s GDP per capita was above the national average, its healthcare resources (e.g., hospital beds, physicians, nurses) per capita were below the national average [20]. Yuhuan established a team-based care model to enhance the management of chronic diseases. A patient with hypertension or diabetes could contract with a family doctor team to receive both preventive and curative care tailored to his or her needs. A family doctor team was multidisciplinary and usually comprised of physicians, nurses, pharmacists, and other healthcare workers who worked in a nearby primary care facility. By the end of 2020, family doctor teams had contracted with 43.57% of residents in Yuhuan. For each contracted resident, the team would be reimbursed with 120 RMB/year (1 RMB approximates 0.15 USD) on a per capita basis, and the payment was jointly financed through the patient (24 RMB), the government (48 RMB), and the health insurance program (48 RMB). Also, Yuhuan has consolidated public hospitals with primary care facilities to form an integrated care delivery system, as required by a national policy aiming at integrating healthcare services locally. Efforts have been made to facilitate information sharing between facilities and to provide technical support for providers at primary care facilities. But residents are still free to visit any hospitals and primary care facilities without a referral. In this study, we hypothesized that higher continuity of care was associated with lower healthcare costs.

## Methods

### Data source and study population

This study was based on a social health insurance claims dataset collected from September 2017 to August 2019 in Yuhuan city in Zhejiang Province, China. This dataset included all 1421 patients with hypertension and/or diabetes who were managed by family doctors through contracts in 8 villages/communities. This claims dataset was initially created to evaluate the impact of a payment reform pilot program implemented in these 8 villages/communities from September 2018 to August 2019. The pilot program was designed for a cost-saving purpose, with no consideration of continuity of care in selecting the sample.

This dataset consolidated claims data of patients covered by the Urban Employee Basic Medical Insurance (UEBMI) or the Resident Basic Medical Insurance (RBMI). Like many other cities in China, these two types of social health insurance programs were available in Yuhuan, in combination covering nearly the entire population in the city. The UEBMI covered all public and private sector’s employees, including those who are self-employed, while the RBMI covered the remaining population. In general, UEBMI provided more generous coverage for its beneficiaries than RBMI. This dataset included patients’ healthcare encounters at all levels and sites of care, primarily in primary care facilities and public hospitals.

### Study design and study population

A cross-sectional study design was adopted to examine the association between continuity of care and healthcare utilization in the same year, using the claims data described above.

The study population included 1406 patients who had at least 2 outpatient encounters from September 2017 to August 2019.

### Measurement

#### Continuity of Care

We calculated four commonly used measures for interpersonal continuity of outpatient care which in this study included outpatient visits, outpatient emergency department visits, and visits to pharmacists [8,21,22]. Detailed formulas for the four continuous variables were presented in Table 1. All of these continuous measures range from 0 to 1, with the value close to 1 representing greater continuity of care.

**Table 1:** The formulas of four continuity of care measures.


CONTINUITY MEASURES	FORMULAS

COC	\frac{{(\mathop \sum \nolimits_{i = 1}^P {n_i}^2) - n}}{{n(n - 1)}}

HI	\mathop \sum \nolimits_{i = 1}^P {\left({\frac{{{{\boldsymbol{n}}_{i}}}}{\boldsymbol{n}}} \right)^2}

UPC	max({\textstyle{{{n_i}} \over n}})

SECON	\frac{{\mathop \sum \nolimits_{j = 1}^{n - 1} {{\boldsymbol{C}}_j}}}{{{\boldsymbol{n}} - 1}}


p = total number of providers; n = total number of visits during the episode; ni = number of visits to provider i; cj = indicator of sequential visits to same providers, equal to 1 if visits j and j+1 are to the same provider, 0 otherwise. COC indicates Bice-Boxerman Continuity of Care Index; HI, Herfindahl Index; SECON, Sequential Continuity Index; UPC, Usual Provider of Care.

1. Bice-Boxerman Continuity of Care (COC) Index.
  COC measures the degree of concentration of care visits, taking into account the visit distribution and the total number of visits [23]. For a given number of total visits, COC tends to increase as the visits are more concentrated in fewer providers. In contrast, for a given visit distribution, COC tends to increase as the total number of visits increases. However, the index is not affected by the sequencing of visits. Higher COC scores indicate higher levels of care concentration which in turn requires lower levels of care coordination.
2. Herfindahl Index (HI)
  As in previous literature examining continuity of care, the Herfindahl Index is calculated by summing the squares of the proportions of visits to all of the providers of a particular patient [24]. HI also measures the degree of care concentration, considering the visit distribution. Like COC, HI is not affected by the sequencing of visits. If a patient’s visits are more concentrated to fewer providers, HI tends to be higher, indicating higher levels of care concentration.
3. Usual provider of care (UPC)
  UPC primarily measures the concentration of visits in the provider most often seen [25]. Its value is not affected by the distribution of visits to other providers or by the sequence of visits. Though measuring a similar concept as HI, UPC only focuses on the density of care from the usual provider, neglecting the remaining providers. Higher scores of UPC indicate higher levels of care concentration in the usual provider of care.
4. Sequential Continuity of Care Index (SECON)
  As the fraction of sequential visit pairs at which the same provider is seen, SECON measures the number of handoffs of information required between providers [26]. SECON is primarily affected by the sequence of visits. For instance, a patient who alternates between two providers will have a score of 0. Higher SECON scores indicate higher levels of continuity from the sequential aspect.

As the identification for each physician was not available in this dataset, continuity was measured at the institutional level.

In addition, to approximate the continuity of primary care, one binary variable was created to indicate that a primary care physician was a patient’s usual provider of care (PCP-UPC). Here, we defined a primary care physician as a physician working in a primary care facility, including township health centers and village clinics in rural areas as well as community health centers and stations in urban areas. This variable was coded as 1 if a patient sought care from a primary care facility most often.

#### Healthcare costs

Two sets of outcome measures were used: (1) costs of all-cause outpatient care, including total outpatient costs, outpatient costs reimbursed by social health insurance and other public funding, and out-of-pocket outpatient costs; and (2) costs of all-cause inpatient care, including total inpatient costs, inpatient costs reimbursed by social health insurance and other public funding, and out-of-pocket inpatient costs. The original dataset was at the encounter level. To generate healthcare costs over the study period, costs of encounters were aggregated at the patient level.

#### Covariates

Covariates in this study were age, sex, socioeconomic status (SES), the village/community, the number of outpatient encounters, and the presence of hypertension and/or diabetes. Whether a patient was covered by UEBMI or RBMI was a proxy for the patient’s SES, as patients with UEBMI were employed or self-employed and had relatively higher SES than those without employment. Whether or not the patient had hypertension and/or diabetes was ascertained by using patients’ medical records in their family doctors’ offices.

### Statistical analysis

We used descriptive statistics and simple bivariate analyses to examine the association between the five continuity measures and patients’ characteristics. We estimated the association between continuity of care and outpatient costs using ordinary least squares (OLS) regression. In addition, we estimated the association between continuity of care and inpatient costs using two-part models: (1) a logistic regression with the presence of any inpatient costs as the dependent variable; and (2) an OLS regression among patients with inpatient costs. All regression models controlled for the aforementioned patient characteristics. A squared term for the number of outpatient encounters was also included in regression models.

These regression analyses were first conducted for the two-year period (from September 2017 to August 2019) with the average yearly costs as the outcomes. The regression analyses were further conducted to examine the association between continuity of care from September 2017 to August 2018 and healthcare costs from September 2018 to August 2019. These analyses aimed at eliminating the potential reverse causality which could not be addressed in a cross-sectional study design [8]. We also conducted stratified analyses by age (those aged 70 years and older vs. those younger than 70), sex (female vs. male), insurance program (UEBMI vs. RBMI), chronic disease (hypertension vs. diabetes vs. both conditions), and the number of outpatient visits (those with 20 outpatient encounters or more vs. those with fewer than 20 outpatient encounters), in order to better account for potential confounding and the heterogeneous impacts of continuity of care across subpopulations.

We reported post-estimation results for regression analyses except for the stratified analyses. For each continuity measure, we predicted the outcomes if the measure was set to 1 from the status quo. Confidence intervals were generated by the bootstrap method using 1000 replications. Regarding outpatient costs, we predicted reduced outpatient costs associated with improved continuity (i.e., if the continuity measure was set to 1 from the status quo) based on OLS models. Regarding inpatient costs, we predicted reduced hospitalization risks, unconditional costs (the net overall costs in all patients), and conditional costs (the costs conditioned on having inpatient costs) associated with improved continuity (as above) based on two-part models.

## Results

### Participant characteristics

Among the 1406 patients, the yearly outpatient cost was on average 2567 RMB. During the two-year study period, 445 patients (31.65%) had been hospitalized, generating a yearly inpatient cost of 3495 RMB among all 1406 patients (unconditional) and 11042 RMB among 445 patients (conditional). The mean value of COC, HI, UPC and SECON were 0.58, 0.61, 0.71, and 0.68, while 43.03% of patients had a PCP-UPC (Table 2). Patients aged 70 years and older tended to have a higher level of continuity than those younger than 70 years of age. The continuity of patients who were covered by RBMI was also higher than those covered by UEBMI. Further, patients with ≥20 outpatient encounters had a lower continuity than other patients (Additional file 1).

**Table 2:** The detailed characteristics of 1406 patients in Yuhuan City between September 2017 and August 2019.


VARIABLES (n = 1406)	N (%)

Age, mean (SD)	65.57 (11.93)

Sex	

Female	752 (53.49)

Male	654 (46.51)

Medical insurance program	

Urban Employee Basic Medical Insurance	140 (9.96)

Resident Basic Medical Insurance	1266 (90.04)

Chronic diseases	

Hypertension	972 (69.13)

Diabetes	116 (8.25)

Both hypertension and diabetes	318 (22.62)

Number of outpatient encounters, mean (SD)	36.19 (32.88)

Village/community	

Village/community 1	140 (9.96)

Village/community 2	162 (11.52)

Village/community 3	265 (18.85)

Village/community 4	217 (15.43)

Village/community 5	85 (6.05)

Village/community 6	214 (15.22)

Village/community 7	137 (9.74)

Village/community 8	186 (13.23)

Total outpatient costs, mean (SD)	2567 (5351)

Reimbursed	1340 (4238)

Out-of-pocket	1226 (1448)

Any inpatient costs, n (%)	445 (31.65)

Total unconditional inpatient costs, mean (SD)	3495 (10461)

Reimbursed	1771 (6457)

Out-of-pocket	1723 (5065)

Total conditional inpatient costs, mean (SD)	11042 (16211)

Reimbursed	5635 (10537)

Out-of-pocket	5445 (7802)

Predictors of interest, mean (SD)	

COC	0.58 (0.24)

HI	0.61 (0.22)

UPC	0.71 (0.19)

SECON	0.68 (0.21)

Having a primary care provider as UPC, n (%)	605 (43.03)


COC indicates Bice-Boxerman Continuity of Care Index; HI, Herfindahl Index; SD, standard deviation; SECON, Sequential Continuity Index; UPC, Usual Provider of Care.

### The association between continuity of care and the outpatient costs

After controlling for covariates, we observed significant negative correlations between continuity of care measures and the yearly outpatient costs. For the four continuous measures of continuity of care, every 0.1-unit increase was associated with 5.87–8.88% lower total costs (151–228 RMB), 3.13–5.95% lower reimbursed costs (42–80 RMB), and 8.88%–12.54% lower out-of-pocket costs (109–154 RMB). Among the 4 indices, UPC and SECON were the most influential measures on outpatient costs. In addition, having a PCP-UPC was associated with 12.50% lower total costs (321 RMB), 1.61% lower reimbursed costs (22 RMB), and 24.42% lower out-of-pocket costs (299 RMB) compared to having no PCP-UPC (Table 3). The full regression results are presented in Additional file 2. Similar patterns were found in the direction and magnitude of associations between continuity of care and outpatient costs across subgroups by age, sex, insurance program, and chronic disease. Notably, the magnitude of associations between continuity of care and outpatient costs was much larger among patients with 20 outpatient encounters or more than those with fewer than 20 outpatient encounters. The results of stratified analyses are presented in Additional files 3–7.

**Table 3:** The association between continuity of care and outpatient costs between September 2017 and August 2019.


PRIMARY PREDICTORS, COEF (95% CI)	COC	HI	UPC	SECON	PCP-UPC

Total costs	–151*** (–208, –94)	–178*** (–240, –116)	–228*** (–298, –158)	–194*** (–259, –130)	–321* (–627, –15)

Reimbursed costs	–42* (–82, –2)	–52 * (–95, –9)	–74** (–123, –25)	–80** (–125, –35)	–22 (–233, 190)

Out-of-pocket costs	–109*** (–132, –86)	–126*** (–151, –101)	–154*** (–182, –126)	–115*** (–141, –88)	–299*** (–424, –174)


*p < 0.05, **p < 0.01, ***p < 0.001. Ordinary least squares models adjusted for age, sex, village, medical insurance program, chronic diseases, number of total outpatient visits, and number of total outpatient visits squared. CI indicates confidence interval; COC, Bice-Boxerman Continuity of Care Index; coef, coefficient; HI, Herfindahl Index; PCP-UPC, Having a primary care provider as the usual provider of care; SECON, Sequential Continuity Index; UPC, Usual Provider of Care.

**Figure 1:**
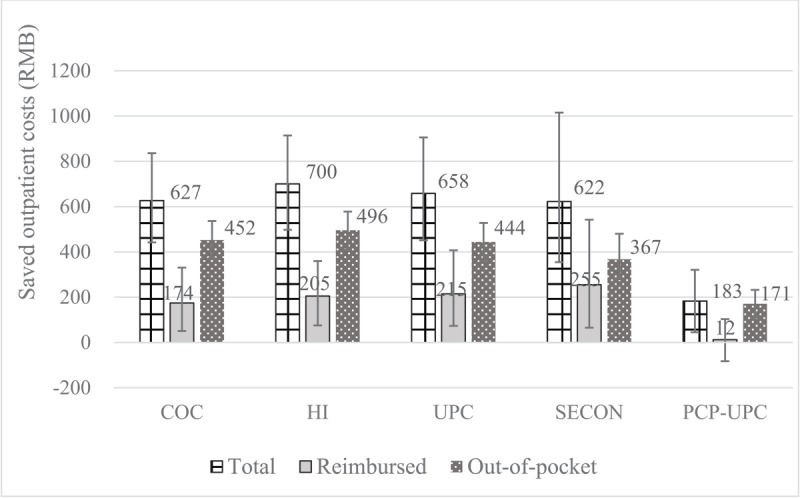
Saved outpatient costs when setting continuity of care to 1 compared to status quo. COC indicates Bice-Boxerman Continuity of Care Index; HI, Herfindahl Index; PCP-UPC, Having a primary care provider as the usual provider of care; SECON, Sequential Continuity Index; UPC, Usual Provider of Care.

Compared with the status quo, if optimum continuity of care measures were to be achieved (each index equals 1), 7.12–27.29% of total outpatient costs (183–700 RMB), 0.92–19.04% of reimbursed costs (12–255 RMB), and 13.91%–40.42% of out-of-pocket costs (171–496 RMB) per patient per year could be saved (Figure 1).

The first-year continuity of care measures were significantly associated with the second-year outpatient costs after controlling for covariates. For the four continuous measures of continuity of care, every 0.1-unit increase was associated with 4.49–7.23% lower total costs (134–215 RMB), 3.16–5.70% lower reimbursed costs (50–91 RMB), and 6.03–8.97% lower out-of-pocket costs (84–124 RMB). UPC had the largest impact on second-year outpatient costs. In comparison with no PCP-UPC, having a PCP-UPC was associated with a reduction of 15.04% in total costs (448 RMB), 8.23% in reimbursed costs (131 RMB), and 22.87% in out-of-pocket costs (317 RMB) (Additional file 8).

Assuming that optimum first-year continuity of care measures were to be reached, 7.47%–21.78% of total second-year outpatient costs (192–559 RMB), 4.19%–18.07% of reimbursed costs (56–242 RMB), and 11.07%–27.59% of out-of-pocket costs (136–338 RMB) per patient could be saved in comparison with the current costs (Additional file 9).

### The association between continuity of care and the inpatient costs

After controlling for covariates, we observed significant negative correlations between continuity of care measures and hospitalization risk, and out-of-pocket conditional inpatient costs. For the four continuous measures of continuity of care, every 0.1-unit increase was associated with a lower likelihood of hospitalization (odds ratio (OR) = 0.74–0.79) and 10.81%–11.91% (589–648 RMB) lower out-of-pocket costs among those who had been hospitalized. Moreover, having a PCP-UPC was associated with a lower likelihood of hospitalization (OR = 0.32) and 46.20% (2515 RMB) lower out-of-pocket costs, compared to having no PCP-UPC (Table 4). The full regression results are presented in Additional files 10 and 11. The magnitude of associations between continuity of care and inpatient costs was larger among women, patients aged 70 and older, patients with RBMI, patients with hypertension, and patients with fewer than 20 outpatient encounters. The results of stratified analyses are presented in Additional files 3–7.

**Table 4:** The association between continuity of care and inpatient costs between September 2017 and August 2019.


PRIMARY PREDICTORS, COEF (95% CI)	COC	HI	UPC	SECON	PCP-UPC

Any cost, OR (95% CI)	0.77*** (0.72, 0.82)	0.74*** (0.69, 0.79)	0.74*** (0.69, 0.80)	0.79*** (0.74, 0.85)	0.32*** (0.24, 0.44)

Total conditional costs (n = 445)	–773 (–1563, 18)	–824 (–1657, 9)	–830 (–1714, 53)	–889* (–1758, –21)	–4887* (–9146, –628)

Reimbursed conditional costs (n = 442)	–168 (–676, 341)	–187 (–722, 349)	–168 (–735, 399)	–277 (–834, 280)	–2286 (–5026, 454)

Out-of-pocket conditional costs (n = 445)	–589** (–971, –206)	–620** (–1023, –217)	–648** (–1076, –221)	–598** (–1019, –177)	–2515* (–4587, –443)


*p < 0.05, **p < 0.01, ***p < 0.001. Ordinary least squares models adjusted for age, sex, village, medical insurance program, chronic diseases, number of total outpatient visits, and number of total outpatient visits squared. CI indicates confidence interval; COC, Bice-Boxerman Continuity of Care Index; coef, coefficient; HI, Herfindahl Index; OR, odds ratio; PCP-UPC, Having a primary care provider as the usual provider of care; SECON, Sequential Continuity Index; UPC, Usual Provider of Care.

**Figure 2:**
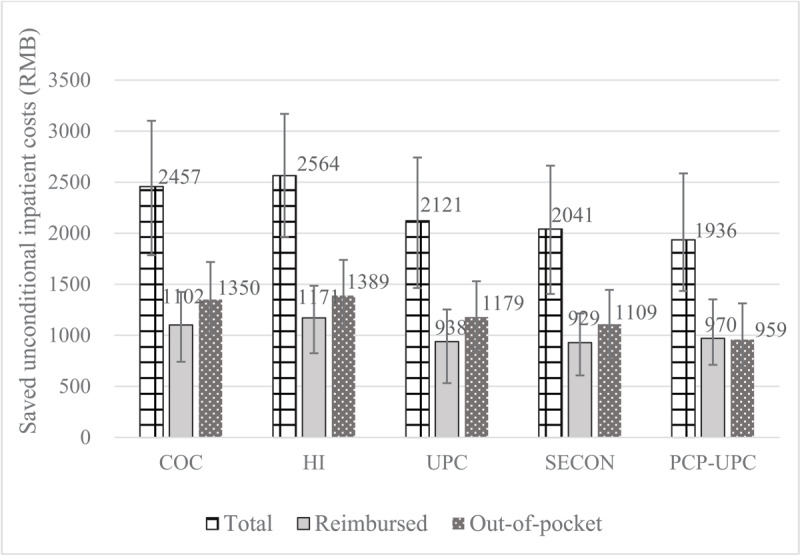
Saved unconditional inpatient costs when setting continuity of care to 1 compared to status quo. COC indicates Bice-Boxerman Continuity of Care Index; HI, Herfindahl Index; PCP-UPC, Having a primary care provider as the usual provider of care; SECON, Sequential Continuity Index; UPC, Usual Provider of Care.

When setting each continuity of care index to 1, hospitalization risk would be reduced by 11.55–18.41% (Additional file 12), while the total, reimbursed, and out-of-pocket unconditional inpatient costs per patient per year can be saved by 55.38–73.35%, 52.43–66.11%, and 55.63–80.61% (1936–2564 RMB, 929–1171 RMB, 959–1389 RMB) (Figure 2) compared to the status quo, respectively.

The first-year continuity of care measures had significant impacts on the second-year hospitalization. For the four continuous measures of continuity of care, every 0.1-unit increase was associated with a lower likelihood of hospitalization (OR = 0.91–0.93). Furthermore, having a PCP-UPC was associated with a lower likelihood of hospitalization (OR = 0.87) compared to no PCP-UPC. Nonetheless, no significant correlation was found between the first-year continuity of care measures and the second-year conditional inpatient costs per patient (Additional file 13).

Post-estimation results showed that the second-year hospitalization risk could be reduced by 0.90–4.38% and total, reimbursed, and out-of-pocket unconditional inpatient costs per patient could be saved by 8.84–40.22% (309–1406 RMB), 15.50–41.08% (274–727 RMB), and 1.74–40.91% (30–705 RMB) respectively, if the first-year continuity of care indices were to increase from the current level to the optimum level (Additional file 14 and 15).

## Discussion

This study is the first to report on continuity of care using claims data in mainland China. We used claims data from Yuhuan city to assess continuity of care and its association with healthcare costs among patients with hypertension and/or diabetes. Continuity measures in this study indicated relatively high levels of continuity of care among chronic disease patients in Yuhuan. Higher continuity was significantly associated with lower healthcare costs in the same year and the second year. When optimum continuity were to be achieved, 7.12–27.29% of total outpatient costs per patient and 55.38–73.35% of total inpatient costs per patient could be saved compared to the status quo during the two-year study period. If optimum continuity were to be achieved in the first year, 7.47%–21.78% of total outpatient costs and 8.84–40.22% of total inpatient costs could be saved in the second-year.

The levels of continuity of care were in the range of what was reported in managed care settings in high-income countries [27,28]. and were higher among certain patient subgroups. Patients aged 70 years and older tended to have a higher level of continuity than their younger counterparts. As the elderly were one of the target populations of family doctor contracting services [15], the elderly might have closer relationships with their family doctors, which in turn boosted continuity of care. This might also explain why patients with RBMI had higher continuity of care than patients with UEBMI: the majority of elders were covered by RBMI as they were no longer employed. In addition, patients with ≥20 outpatient encounters had a lower continuity than other patients. These patients might have more complicated conditions which required healthcare from a diverse set of providers. However, some previous studies showed that patients with more healthcare needs valued continuity of care more and experienced greater continuity of care [29,30]. This contrast suggested that more efforts are warranted to enhance the continuity of care among patients with complex healthcare needs.

The associations we observed in our study between continuity of care and reduced healthcare costs and decreased risks of hospitalization were consistent with the previous studies, though the contexts differed [31,32]. Like other parts of China (except for Shenzhen and Dongguan city) [33], patients in Yuhuan have direct access to specialty care. However, gatekeeping is common in tax-funded health systems, social health insurance systems, and managed care settings in high-income countries. As gate-keeping could enhance continuity of care and reduce healthcare costs [34], it could possibly confound the relationship between continuity of care and healthcare costs. In this study, we ascertained the association between continuity of care and healthcare costs even without formal gate-keeping and referral mechanisms. Notably, our findings revealed that out-of-pocket costs were particularly sensitive to continuity of care. In China, the incidence of catastrophic health expenditure was as high as 15–20.3% [35,36,37]. Previous literature on catastrophic health expenditure has focused on the financial protection effects of social health insurance programs in China [38]. Our findings suggested that efforts to improve continuity of care through the delivery system reform might be another way to alleviate the risk of catastrophic health expenditure. Furthermore, stratified analyses suggested that the impacts of continuity of care on healthcare costs were even larger among vulnerable populations, such as women, older patients, and patients with lower socioeconomic status. These findings suggested that interventions to promote continuity of care might also be meaningful to improve health equity.

This study highlighted the role of primary care in providing continuous care in China. China’s primary care system faced a number of challenges to combat the growing disease burden of non-communicable chronic diseases, including the lack of well-trained human resources, fragmented health information systems, and distorting financial incentives [39]. The efficiency of primary health facilities in care provision has been questioned [40,41]. We found that having a primary care provider as the usual source of care was associated with lower outpatient costs and hospitalization risks which in turn reduced inpatient costs. These findings suggested that for patients with direct access to specialty care, those who concentrated their care more in primary care settings might have lower costs and better outcomes. These findings underscored the potential role of primary care providers (e.g., family doctor teams) in enhancing the continuity of care as experienced by patients, particularly for patients with chronic diseases.

This study has several limitations. First, as an observational study using claims data, the reported associations might not imply causality. We cannot rule out the possibility of uncontrolled confoundings, such as patients’ socioeconomic status for which we used attendance of different types of health insurance programs as a proxy. We also conducted stratified analyses by age, sex, insurance program, chronic disease, and the number of outpatient visits to better account for potential confounding. However, due to data limitations, we were not able to adjust for comorbidities other than hypertension and diabetes. We acknowledged that patients with more healthcare needs might have lower continuity of care and higher healthcare costs. In this scenario (the confounder-exposure association and the confounder-outcome association are in opposite directions), the bias should be towards the null. Second, we were not able to compare provider-level continuity of care to institutional-level continuity of care, as the provider’s identification was unavailable. As facilities usually had more than one provider, continuity of care at the provider level should be lower than but highly correlated with continuity of care at the institutional level [42]. Third, also due to data limitations, we were not able to differentiate the purposes of outpatient encounters. By including all the outpatient encounters, instead of the outpatient encounters only related to chronic disease management, we tended to underestimate the continuity of care. Furthermore, the findings might not be generable to other parts of China and/or to the population without hypertension or diabetes. However, Yuhuan is a city with below-average healthcare resources per capita in China. Family doctor contracting services and other local efforts to enhance care integration were all required by national policies. Thus, to some extent, our findings may still be relevant to other parts of China.

In conclusion, we found relatively high continuity of care among the study population, and care continuity indicators were consistently associated with reduced healthcare costs and decreased risks of hospitalization. Future health reform should focus on improving healthcare continuity, in order to achieve a people-centered integrated care system.

## Data Accessibility Statement

The datasets generated and/or analyzed during the current study are not publicly available due to individual privacy protection but are available from the corresponding author on reasonable request.

## Additional Files

The additional files for this article can be found as follows:

10.5334/ijic.5994.s1 Additional file 1. Table which demonstrates bivariate analyses of continuity of care and patients’ characteristics.docx.

10.5334/ijic.5994.s2 Additional file 2. Table which presents the full regression results of the outpatient costs.docx.

10.5334/ijic.5994.s3 Additional file 3. Table which presents the subgroup analyses of association between continuity of care measures and outpatient/inpatient costs based on age.docx.

10.5334/ijic.5994.s4 Additional file 4. Table which presents the subgroup analyses of association between continuity of care measures and outpatient/inpatient costs based on gender.docx.

10.5334/ijic.5994.s5 Additional file 5. Table which presents the subgroup analyses of association between continuity of care measures and outpatient/inpatient costs based on medical insurance program.docx.

10.5334/ijic.5994.s6 Additional file 6. Table which presents the subgroup analyses of association between continuity of care measures and outpatient/inpatient costs based on disease type.docx.

10.5334/ijic.5994.s7 Additional file 7. Table which presents the subgroup analyses of association between continuity of care measures and outpatient/inpatient costs based on number of visits.docx.

10.5334/ijic.5994.s8 Additional file 8. Table which presents the association between the first-year continuity of care and the second-year outpatient costs.docx.

10.5334/ijic.5994.s9 Additional file 9. Figure which demonstrates saved second-year outpatient costs when setting the first-year continuity of care to 1 compared to status quo.docx.

10.5334/ijic.5994.s10 Additional file 10. Table which presents the full regression results of the hospitalization risk.docx.

10.5334/ijic.5994.s11 Additional file 11. Table which presents the full regression results of the conditional inpatient costs.docx.

10.5334/ijic.5994.s12 Additional file 12. Figure which demonstrates reduced hospitalization risk when setting continuity of care measures to 1 compared to status quo.docx.

10.5334/ijic.5994.s13 Additional file 13. Table which presents the association between the first-year continuity of care and the second-year inpatient costs.docx.

10.5334/ijic.5994.s14 Additional file 14. Figure which presents reduced second-year hospitalization risk when setting the first-year continuity of care to 1 compared to status quo.docx.

10.5334/ijic.5994.s15 Additional file 15. Figure which presents saved second-year unconditional inpatient costs when setting the first-year continuity of care to 1 compared to status quo.docx.

## Abbreviations

COC: Bice-Boxerman Continuity of Care Index; HI: Herfindahl Index; SECON: Sequential Continuity of Care Index; UPC: Usual Provider of Care; LMICs: low- and middle-income countries; NCDs: non-communicable diseases; PCIC: People-Centered Integrated Care; UEBMI: Urban Employee Basic Medical Insurance; RBMI: Resident Basic Medical Insurance; PCP-UPC: a primary care physician was a patient’s usual provider of care; SES: socioeconomic status; OLS: ordinary least squares; SD: standard deviation; CI: confidence interval; coef: coefficient; OR: odds ratio.
