# Supplementary material for: Continuity of Care and Healthcare Costs among Patients with Chronic Disease: Evidence from Primary Care Settings in China
Source: Int J Integr Care. 2022 Oct 12;22(4):4. doi: 10.5334/ijic.5994 (PMC9562970; doi:10.5334/ijic.5994)
Supplement: Additional file 1. — Table which demonstrates bivariate analyses of continuity of care and patients’ characteristics.docx. [file ijic-22-4-5994-s1.pdf]

Additional file 1. Bivariate analyses of continuity of care and patients' characteristics among 1406 patients in Yuhuan City between September 2017 and August 2019

Variables	COC	HI	UPC	SECON	PCP-UPC
Total	0.58	0.61	0.71	0.68	0.43
Sex					
Female	0.58	0.60	0.71	0.68	0.45
Male	0.59	0.61	0.72	0.68	0.41
Age					
<60	0.57	0.59	0.70	0.66	0.37
60-69	0.58	0.60	0.71	0.67	0.41
≥70	0.60	0.63*	0.73	0.71**	0.50***
Socioeconomic status					
Urban Employee Basic Medical Insurance	0.48	0.49	0.61	0.56	0.15
Resident Basic Medical Insurance	0.60***	0.62***	0.72***	0.69***	0.46***
Chronic diseases					
Having Hypertension only	0.58	0.61	0.71	0.69	0.44
Having diabetes only	0.57	0.59	0.70	0.66	0.41
Having both hypertension and diabetes	0.59	0.61	0.71	0.67	0.42
Number of outpatient encounters					
<10	0.57	0.66	0.73	0.64	0.70
10-19	0.56	0.59	0.69	0.67	0.62
≥20	0.59	0.60**	0.71	0.69	0.35***

*p<0.05, **p<0.01, ***p<0.001

COC indicates Bice-Boxerman Continuity of Care Index; HI, Herfindahl Index; PCP-UPC, Having a primary care provider as the usual provider of care; SECON, Sequential Continuity Index; UPC, Usual Provider of Care.
