# Supplementary material for: Continuity of Care and Healthcare Costs among Patients with Chronic Disease: Evidence from Primary Care Settings in China
Source: Int J Integr Care. 2022 Oct 12;22(4):4. doi: 10.5334/ijic.5994 (PMC9562970; doi:10.5334/ijic.5994)
Supplement: Additional file 2. — Table which presents the full regression results of the outpatient costs.docx. [file ijic-22-4-5994-s2.pdf]

Additional file 2. The full regression results of the outpatient costs among 1406 patients in Yuhuan City between September 2017 and August 2019

**Total**	coef（95% CI）				
	COC	HI	UPC	SECON	PCP-UPC
Continuity of care measures	-151*** (-208,-94)	-178*** (-240,-116)	-228*** (-298,-158)	-194*** (-259,-130)	-321* (-627,-15)
Sex	-29 (-292,234)	-32 (-294,230)	-33 (-294,229)	-17 (-279,244)	8 (-257,272)
Age	-10 (-21,1)	-10 (-21,1)	-10 (-21,1)	-9 (-20,2)	-10 (-22,1)
Village/community					
1	1109*** (539,1680)	1076*** (506,1646)	1051*** (482,1619)	1162*** (597,1728)	1477*** (896,2058)
2	-55 (-565,454)	-91 (-601,419)	-136 (-644,373)	-51 (-557,455)	204 (-300,708)
3	104 (-434,642)	67 (-471,604)	31 (-505,566)	73 (-463,608)	517 (-27,1062)
4	962** (285,1639)	911** (234,1589)	882* (207,1557)	1007** (336,1678)	1423*** (734,2113)
5	-54 (-571,464)	-50 (-567,467)	-56 (-571,459)	-33 (-549,484)	-47 (-569,475)
6	813** (219,1407)	781** (187,1374)	743* (152,1335)	834** (244,1423)	1223*** (618,1828)
7	-347 (-882,188)	-360 (-895,174)	-371 (-904,162)	-326 (-859,208)	-203 (-745,338)
Having Hypertension only	-545*** (-857,-232)	-542*** (-854,-230)	-541*** (-852,-230)	-498** (-809,-187)	-520** (-835,-205)
Having diabetes only	133 (-394,659)	138 (-387,664)	129 (-395,653)	159 (-366,684)	174 (-356,705)
Resident Basic Medical Insurance	-1208*** (-1707,-709)	-1203*** (-1701,-705)	-1178*** (-1675,-682)	-1158*** (-1657,-659)	-1340*** (-1840,-840)
Number of outpatient encounters	48*** (40,56)	46*** (38,54)	47*** (39,55)	49*** (41,56)	49*** (41,57)
Number of outpatient encounters	0*** (0,0)	0*** (0,0)	0*** (0,0)	0*** (0,0)	0*** (0,0)

squared					
Constant	3023*** (1890,4156)	3296*** (2142,4450)	3836*** (2636,5035)	3280*** (2136,4424)	2107*** (1027,3187)

**Reimbursed**	coef（95% CI）				
	COC	HI	UPC	SECON	PCP-UPC
Continuity of care measures	-42* (-82,-2)	-52* (-95,-9)	-74** (-123,-25)	-80*** (-125,-35)	-22 (-233,190)
Sex	-53 (-236,131)	-54 (-237,129)	-56 (-239,127)	-54 (-237,128)	-40 (-223,143)
Age	-8 (-15,0)	-8 (-15,0)	-8 (-15,0)	-7 (-15,1)	-8 (-16,0)*
Village/community					
1	386 (-12,783)	372 (-26,770)	355 (-42,752)	376 (-18,770)	461 (58,863)*
2	-150 (-505,205)	-164 (-520,191)	-189 (-544,167)	-183 (-535,170)	-78 (-426,271)
3	-105 (-479,270)	-120 (-495,255)	-142 (-517,232)	-155 (-528,218)	-17 (-394,361)
4	521* (49,993)	502* (29,974)	480* (8,952)	499* (32,967)	616* (139,1093)
5	-186 (-547,175)	-185 (-545,176)	-186 (-546,174)	-176 (-536,184)	-188 (-549,174)
6	190 (-223,604)	177 (-237,591)	155 (-258,569)	164 (-247,574)	275 (-144,693)
7	-248 (-620,125)	-253 (-625,120)	-259 (-632,113)	-250 (-621,122)	-220 (-595,155)
Having Hypertension only	-224* (-442,-6)	-223* (-441,-6)	-224* (-441,-7)	-209 (-426,8)	-216 (-434,2)
Having diabetes only	150 (-216,517)	151 (-215,518)	147 (-219,513)	155 (-210,521)	163 (-205,530)
Resident Basic Medical Insurance	-1986*** (-2334,-1639)	-1983*** (-2330,-1635)	-1970*** (-2317,-1623)	-1946*** (-2294,-1599)	-2027*** (-2373,-1681)
Number of outpatient encounters	11*** (6,17)	11*** (5,16)	11*** (5,16)	11*** (5,16)	12*** (6,17)
Number of outpatient encounters squared	0*** (0,0)	0*** (0,0)	0*** (0,0)	0*** (0,0)	0*** (0,0)
Constant	3032*** (2243,3821)	3126*** (2321,3932)	3344*** (2506,4183)	3268*** (2471,4065)	2762*** (2014,3509)

**Out-of-pocket**	coef（95% CI）

	COC	HI	UPC	SECON	PCP-UPC
Continuity of care measures	-109*** (-132,-86)	-126*** (-151,-101)	-154*** (-182,-126)	-115*** (-141,-88)	-299*** (-424,-174)
Sex	24 (-82,130)	22 (-83,128)	24 (-81,128)	37 (-69,143)	47 (-61,155)
Age	-3 (-7,2)	-2 (-7,2)	-3 (-7,2)	-2 (-7,3)	-2 (-7,2)
Village/community					
1	724*** (494,953)	704*** (475,933)	696*** (468,923)	787*** (558,1016)	1016*** (779,1254)
2	95 (-110,300)	73 (-131,278)	53 (-151,257)	132 (-73,337)	282 (76,488)**
3	208 (-8,425)	186 (-30,402)	173 (-42,387)	228* (11,445)	534*** (311,756)
4	441** (169,714)	410** (138,682)	402** (131,672)	508*** (236,780)	807*** (526,1089)
5	132 (-76,341)	135 (-73,342)	130 (-76,337)	143 (-66,353)	141 (-72,354)
6	622*** (383,861)	603*** (365,842)	588*** (351,825)	670*** (431,909)	948*** (701,1195)
7	-100 (-315,116)	-108 (-322,107)	-112 (-325,102)	-76 (-292,140)	17 (-204,238)
Having Hypertension only	-321*** (-447,-195)	-318*** (-444,-193)	-317*** (-441,-192)	-289*** (-416,-163)	-304*** (-433,-176)
Having diabetes only	-18 (-230,194)	-13 (-224,198)	-18 (-228,192)	4 (-209,217)	11 (-205,228)
Resident Basic Medical Insurance	779*** (578,980)	780*** (580,980)	791*** (593,990)	788*** (586,991)	687*** (483,892)
Number of outpatient encounters	37*** (34,40)	36*** (32,39)	36*** (33,39)	38*** (34,41)	37*** (34,40)
Number of outpatient encounters squared	0** (0,0)	0 (0,0)	0* (0,0)	0** (0,0)	0** (0,0)
Constant	-9 (-465,447)	170 (-294,633)	491* (11,972)	12 (-452,476)	-655** (-1095,-214)

CI indicates confidence interval; COC, Bice-Boxerman Continuity of Care Index; Coef, coefficient; HI, Herfindahl Index; PCP-UPC, Having a primary care provider as the usual provider of care; SECON, Sequential Continuity Index; UPC, Usual

Provider of Care.
