# Supplementary material for: Continuity of Care and Healthcare Costs among Patients with Chronic Disease: Evidence from Primary Care Settings in China
Source: Int J Integr Care. 2022 Oct 12;22(4):4. doi: 10.5334/ijic.5994 (PMC9562970; doi:10.5334/ijic.5994)
Supplement: Additional file 3. — Table which presents the subgroup analyses of association between continuity of care measures and outpatient/inpatient costs based on age.docx. [file ijic-22-4-5994-s3.pdf]

Additional file 3. The subgroup analyses of association between continuity of care measures and outpatient/inpatient costs based on age.

**Primary predictors, coef (95% CI)**	**COC**	**HI**	**UPC**	**SECON**	**PCP-UPC**
Association between continuity of care measures and outpatient costs					
Subgroup: age≥ 70 years old (N=504)					
Total outpatient costs	-120*** (-179,-62)	-143*** (-207,-79)	-178*** (-251,-105)	-128*** (-198,-59)	-451** (-769,-133)
Reimbursed outpatient costs	-24 (-56,9)	-28 (-64,7)	-37 (-77,4)	-17 (-56,21)	-96 (270,79)
Out-of-pocket outpatient costs	-97*** (-131,-63)	-115*** (-152,-78)	-141*** (-184,-99)	-111*** (-152,-71)	-355*** (-542,-169)
Subgroup: age< 70 years old (N=902)					
Total outpatient costs	-172*** (-256,-89)	-197*** (-287,-107)	-262*** (-363,-160)	-237*** (-329,-146)	-174 (-619,270)
Reimbursed outpatient costs	-56 (-115,4)	-65* (-130,0)	-99** (-172,-26)	-119*** (-185,-53)	65 (-252,382)
Out-of-pocket outpatient costs	-117*** (-147,-86)	-132*** (-165,-99)	-163*** (-200,-126)	-119*** (-153,-85)	-240** (-406,-74)
Association between continuity of care measures and inpatient costs					
Subgroup: age≥ 70 years old					
Any inpatient cost, OR (95% CI) (N=504)	0.78*** (0.71,0.84)	0.75*** (0.69,0.82)	0.75*** (0.68,0.83)	0.80*** (0.73,0.87)	0.38*** (0.25,0.57)
Total conditional inpatient costs (N =214)	-695 (-1848,458)	-732 (-1953,490)	-702 (-2025,622)	-422 (-1789,945)	-7312* (-13917,-707)
Reimbursed conditional inpatient costs (N =213)	62 (-782,907)	79 (-816,974)	121 (-844,1086)	100 (-895,1094)	-3222 (-8115,1671)
Out-of-pocket conditional inpatient costs (N =214)	-718** (-1221,-215)	-769** (-1302,-237)	-787** (-1365,-209)	-486 (-1089,116)	-3910** (-6821,-998)
Subgroup: age< 70 years old					
Any inpatient cost, OR (95% CI) (N=902)	0.78*** (0.71,0.84)	0.75*** (0.69,0.82)	0.75*** (0.68,0.83)	0.80*** (0.73,0.87)	0.38*** (0.25,0.57)
Total conditional inpatient costs (N= 231)	-595 (-1732,541)	-586 (-1775,604)	-701 (-1928,526)	-1125 (-2315,66)	-2021 (-8141,4099)
Reimbursed conditional	-244	-241	-279	-488	-1456

inpatient costs (N= 229)	(-838,350)	(-863,381)	(-920,363)	(-1111,134)	(-4645,1732)
Out-of-pocket conditional inpatient costs (N= 231)	-354<br>(-961,254)	-347<br>(-983,289)	-426<br>(-1081,230)	-629<br>(-1265,7)	-604<br>(-3879,2671)

*p<0.05, **p<0.01, ***p<0.001.

Ordinary least squares models adjusted for age, sex, village, medical insurance program, chronic diseases, number of total outpatient visits, number of total outpatient visits squared.

CI indicates confidence interval; COC, Bice-Boxerman Continuity of Care Index; coef, coefficient; HI, Herfindahl Index; PCP-UPC, Having a primary care provider as the usual provider of care; SECON, Sequential Continuity Index; UPC, Usual Provider of Care.
