# Supplementary material for: Continuity of Care and Healthcare Costs among Patients with Chronic Disease: Evidence from Primary Care Settings in China
Source: Int J Integr Care. 2022 Oct 12;22(4):4. doi: 10.5334/ijic.5994 (PMC9562970; doi:10.5334/ijic.5994)
Supplement: Additional file 4. — Table which presents the subgroup analyses of association between continuity of care measures and outpatient/inpatient costs based on gender.docx. [file ijic-22-4-5994-s4.pdf]

Additional file 4. The subgroup analyses of association between continuity of care measures and outpatient/inpatient costs based on gender.

**Primary predictors, coef (95% CI)**	**COC**	**HI**	**UPC**	**SECON**	**PCP-UPC**
Association between continuity of care measures and outpatient costs					
Subgroup: female (N= 752)					
Total outpatient costs	-134*** (-207,-60)	-156*** (-237,-75)	-207*** (-297,-116)	-158*** (-242,-75)	-298 (-698,103)
Reimbursed outpatient costs	-35 (-86,16)	-42 (-98,14)	-66* (-129,-3)	-61* (-119,-3)	8 (-267,283)
Out-of-pocket outpatient costs	-99*** (-128,69)	-114*** (-146,-82)	-140*** (-176,-104)	-97*** (-131,64)	-306*** (-467,-145)
Subgroup: male (N= 654)					
Total outpatient costs	-207*** (-296,-119)	-240*** (-335,-145)	-292*** (-400,-184)	-270*** (-370,-170)	-411 (-880,57)
Reimbursed outpatient costs	-74* (-136,12)	-89** (-155,22)	-110** (-186,-34)	-122*** (-192,-51)	-93 (-420,234)
Out-of-pocket outpatient costs	-133*** (-169,-97)	-151*** (-190,-113)	-182*** (-226,-139)	-148*** (-189,-107)	-319** (-513,-125)
Association between continuity of care measures and inpatient costs					
Subgroup: female					
Any inpatient cost, OR (95% CI) (N=752)	0.76*** (0.70,0.83)	0.73*** (0.66,0.80)	0.75*** (0.68,0.82)	0.79*** (0.73,0.87)	0.27*** (0.17,0.41)
Total conditional inpatient costs (N = 240)	-1465** (-2448,-482)	-1598** (-2636,-559)	-1556** (-2638,-475)	-1153* (-2196,-109)	-6222* (-11218,1227)
Reimbursed conditional inpatient costs (N = 239)	-532* (-987,-78)	-588* (-1069,108)	-579* (-1076,-82)	-366 (-846,114)	-2636* (-4937,-334)
Out-of-pocket conditional inpatient costs (N = 240)	-902** (-1501,303)	-977** (-1610,344)	-949** (-1609,290)	-758* (-1393,122)	-3421* (-6475,-367)
Subgroup: male					
Any inpatient cost, OR	0.77***	0.75***	0.73***	0.78***	0.39***

(95% CI) (N= 654)	(0.71,0.85)	(0.68,0.83)	(0.66,0.82)	(0.71,0.86)	(0.25,0.62)
Total conditional inpatient costs (N= 205)	-352<br>(-1660,956)	-354<br>(-1726,1019)	-360<br>(-1834,1114)	-683<br>(-2152,786)	-3794<br>(-11323,3735)
Reimbursed conditional inpatient costs (N= 203)	12<br>(-941,965)	15<br>(-985,1015)	50<br>(-1024,1124)	-268<br>(-1337,801)	-1941<br>(-7418,3536)
Out-of-pocket conditional inpatient costs (N= 205)	-367<br>(-876,142)	-372<br>(-907,163)	-414<br>(-988,160)	-417<br>(-990,156)	-1854<br>(-4796,1087)

*p<0.05, **p<0.01, ***p<0.001.

Ordinary least squares models adjusted for age, village, medical insurance program, chronic diseases, number of total outpatient visits, number of total outpatient visits squared.

CI indicates confidence interval; COC, Bice-Boxerman Continuity of Care Index; coef, coefficient; HI, Herfindahl Index; PCP-UPC, Having a primary care provider as the usual provider of care; SECON, Sequential Continuity Index; UPC, Usual Provider of Care.
