# Supplementary material for: Continuity of Care and Healthcare Costs among Patients with Chronic Disease: Evidence from Primary Care Settings in China
Source: Int J Integr Care. 2022 Oct 12;22(4):4. doi: 10.5334/ijic.5994 (PMC9562970; doi:10.5334/ijic.5994)
Supplement: Additional file 5. — Table which presents the subgroup analyses of association between continuity of care measures and outpatient/inpatient costs based on medical insurance program.docx. [file ijic-22-4-5994-s5.pdf]

Additional file 5. The subgroup analyses of association between continuity of care measures and outpatient/inpatient costs based on medical insurance program.

**Primary predictors, coef (95% CI)**	**COC**	**HI**	**UPC**	**SECON**	**PCP-UPC**
Association between continuity of care measures and outpatient costs					
Subgroup: Resident Basic Medical Insurance (N=1,266)					
Total outpatient costs	-142*** (-199,-85)	-169*** (-231,-107)	-220*** (-290,-149)	-190*** (-255,-125)	-274 (-577,30)
Reimbursed outpatient costs	-35 (-75,4)	-45* (-88,-2)	-67** (-116,18)	-78*** (-123,33)	26 (-182,234)
Out-of-pocket outpatient costs	-106*** (-129,-84)	-124*** (-148,-100)	-152*** (-180,-125)	-112*** (-138,-86)	-300*** (-421,-179)
Subgroup: Employee Basic Medical Insurance (N=140)					
Total outpatient costs	-253 (-540,35)	-272 (-565,21)	-307* (-612,-2)	-224 (-544,95)	-1077 (-2788,634)
Reimbursed outpatient costs	-125 (-343,92)	-140 (-361,82)	-144 (-376,87)	-83 (-324,158)	-1030 (-2310,250)
Out-of-pocket outpatient costs	-128* (-249,-6)	-132* (-256,-8)	-163* (-291,-34)	-141* (-276,-7)	-47 (-780,685)
Association between continuity of care measures and inpatient costs					
Subgroup: Resident Basic Medical Insurance					
Any inpatient cost, OR (95% CI) (N=1,266)	0.76*** (0.71,0.81)	0.73*** (0.68,0.79)	0.73*** (0.67,0.78)	0.78*** (0.73,0.84)	0.32*** (0.24,0.45)
Total conditional inpatient costs (N =398)	-1154*** (-1831,-478)	-1201** (-1913,-488)	-1202** (-1963,-441)	-1126** (-1863,-388)	-4896** (-8436,-1355)
Reimbursed conditional inpatient costs (N =395)	-469** (-778,-161)	-484** (-809,-159)	-481** (-827,-134)	-458** (-793,-123)	-2079* (-3692,-466)
Out-of-pocket conditional inpatient costs (N =398)	-672** (-1078,265)	-702** (-1130,274)	-708** (-1166,251)	-653** (-1096,210)	-2737* (-4865,-608)
Subgroup: Employee Basic Medical Insurance					
Any inpatient cost, OR	0.79	0.79	0.83	0.81	0.30

(95% CI) (N=140)	(0.62,1.02)	(0.62,1.02)	(0.65,1.07)	(0.62,1.05)	(0.05,1.74)
Total conditional inpatient costs (N=47)	1006 (-5651,7663)	1011 (-5791,7814)	540 (-6340,7421)	320 (-7579,8220)	-10474 (-70133,49186)
Reimbursed conditional inpatient costs (N=47)	1168 (-4287,6624)	1174 (-4400,6749)	977 (-4661,6616)	617 (-5863,7097)	-10695 (-59604,38215)
Out-of-pocket conditional inpatient costs (N=47)	-162 (-1799,1474)	-163 (-1836,1509)	-437 (-2121,1247)	-297 (-2235,1641)	221 (-14464,14905)

*p<0.05, **p<0.01, ***p<0.001.

Ordinary least squares models adjusted for age, sex, village, chronic diseases, number of total outpatient visits, number of total outpatient visits squared.

CI indicates confidence interval; COC, Bice-Boxerman Continuity of Care Index; coef, coefficient; HI, Herfindahl Index; PCP-UPC, Having a primary care provider as the usual provider of care; SECON, Sequential Continuity Index; UPC, Usual Provider of Care.
