# Supplementary material for: Continuity of Care and Healthcare Costs among Patients with Chronic Disease: Evidence from Primary Care Settings in China
Source: Int J Integr Care. 2022 Oct 12;22(4):4. doi: 10.5334/ijic.5994 (PMC9562970; doi:10.5334/ijic.5994)
Supplement: Additional file 6. — Table which presents the subgroup analyses of association between continuity of care measures and outpatient/inpatient costs based on disease type.docx. [file ijic-22-4-5994-s6.pdf]

Additional file 6. The subgroup analyses of association between continuity of care measures and outpatient/inpatient costs based on disease type.

**Primary predictors, coef (95% CI)**	**COC**	**HI**	**UPC**	**SECON**	**PCP-UPC**
Association between continuity of care measures and outpatient costs					
Subgroup: patients only had hypertension (N=972)					
Total costs	-140*** (-206,-73)	-166*** (-239,-93)	-223*** (-306,-140)	-200*** (-275,-124)	-309 (-673,55)
Reimbursed costs	-43 (-91,4)	-53* (-105,-1)	-81** (-141,-21)	-99*** (-153,-44)	8 (-252,267)
Out-of-pocket costs	-96*** (-120,-72)	-113*** (-139,-87)	-142*** (-171,-113)	-101*** (-128,-74)	-317*** (-448,-185)
Subgroup: patients only had diabetes (N=116)					
Total costs	-300* (-542,-57)	-332* (-594,-70)	-447** (-733,-162)	-363** (-623,-103)	-281 (-1521,960)
Reimbursed costs	-155 (-325,16)	-170 (-354,14)	-249* (-450,-48)	-199* (-381,-16)	-249 (-1109,610)
Out-of-pocket costs	-145** (-246,-43)	-162** (-272,-53)	-199** (-318,-79)	-165** (-273,-56)	-31 (-555,492)
Subgroup: patients had both conditions (N=318)					
Total costs	-163** (-280,-45)	-177** (-299,-54)	-191** (-327,-54)	-115 (-248,18)	-483 (-1078,112)
Reimbursed costs	-10 (-82,61)	-14 (-89,60)	-6 (-89,77)	32 (-48,112)	-110 (-469,249)
Out-of-pocket costs	-152*** (-217,-88)	-162*** (-230,-95)	-184*** (-259,-109)	-147*** (-220,-74)	-373* (-706,-40)
Association between continuity of care measures and inpatient costs					
Subgroup: patients only had hypertension					
Any cost, OR (95% CI) (N=972)	0.76*** (0.70,0.82)	0.72*** (0.66,0.78)	0.72*** (0.66,0.79)	0.78*** (0.72,0.84)	0.31*** (0.21,0.45)
Total conditional costs (N=283)	-890* (-1734,-47)	-932* (1826,-38)	-1171* (-2116,-225)	-816 (-1731,99)	-3859 (-8353,635)

Reimbursed conditional costs (N=282)	-255 (-689,179)	-266 (-726,194)	-341 (-828,147)	-210 (-680,260)	-1961 (-4259,337)
Out-of-pocket conditional costs (N=283)	-640** (-1123,-157)	-671* (-1183,-159)	-836** (-1377,-296)	-605* (-1129,-80)	-1895 (-4484,694)
Subgroup: patients only had diabetes					
Any cost, OR (95% CI) (N=116)	0.86 (0.69,1.07)	0.83 (0.66,1.05)	0.82 (0.64,1.05)	0.93 (0.74,1.16)	0.49 (0.17,1.41)
Total conditional costs (N=38)	-1131 (-3559,1298)	-1206 (-3732,1321)	-1188 (-3867,1492)	-1329 (-4012,1355)	-1932 (-16276,12411)
Reimbursed conditional costs (N=37)	-1172 (-2390,46)	-1233 (-2499,33)	-1361* (-2707,-15)	-1403* (-2733,-74)	-1783 (-9415,5850)
Out-of-pocket conditional costs (N=38)	-89 (-1447,1270)	-110 (-1524,1305)	-28 (-1525,1469)	-74 (-1580,1431)	352 (-7538,8242)
Subgroup: patients had both conditions					
Any cost, OR (95% CI) (N=318)	0.77*** (0.68,0.89)	0.76*** (0.66,0.88)	0.76*** (0.65,0.89)	0.78*** (0.67,0.90)	0.31*** (0.16,0.61)
Total conditional costs (N=124)	18 (-2095,2131)	33 (-2168,2234)	311 (-2024,2647)	-493 (-2922,1935)	-6718 (-18057,4620)
Reimbursed conditional costs (N=123)	606 (-962,2175)	643 (-990,2276)	747 (-972,2467)	198 (-1596,1991)	-2380 (-10924,6163)
Out-of-pocket conditional costs (N=124)	-478 (-1303,347)	-497 (-1356,362)	-347 (-1262,568)	-612 (-1559,335)	-3896 (-8315,523)

*$p<0.05$, **$p<0.01$, ***$p<0.001$.

Ordinary least squares models adjusted for age, sex, village, medical insurance program, number of total outpatient visits, number of total outpatient visits squared.

CI indicates confidence interval; COC, Bice-Boxerman Continuity of Care Index; coef, coefficient; HI, Herfindahl Index; PCP-UPC, Having a primary care provider as the usual provider of care; SECON, Sequential Continuity Index; UPC, Usual Provider of Care.
