# Supplementary material for: Continuity of Care and Healthcare Costs among Patients with Chronic Disease: Evidence from Primary Care Settings in China
Source: Int J Integr Care. 2022 Oct 12;22(4):4. doi: 10.5334/ijic.5994 (PMC9562970; doi:10.5334/ijic.5994)
Supplement: Additional file 7. — Table which presents the subgroup analyses of association between continuity of care measures and outpatient/inpatient costs based on number of visits.docx. [file ijic-22-4-5994-s7.pdf]

Additional file 7. The subgroup analyses of association between continuity of care measures and outpatient/inpatient costs based on number of visits.

**Primary predictors, coef (95% CI)**	**COC**	**HI**	**UPC**	**SECON**	**PCP-UPC**
Association between continuity of care measures and outpatient costs					
Subgroup: number of visits >=20 (N=1,023)					
Total outpatient costs	-236*** (-324,-148)	-247*** (-337,-156)	-325*** (-427,-223)	-349*** (-453,-246)	-343 (-765,79)
Reimbursed outpatient costs	-71* (-133,10)	-75* (-139,12)	-112** (-184,-40)	-150*** (-223,-77)	-27 (-320,267)
Out-of-pocket outpatient costs	-165*** (-199,-130)	-171*** (-207,-136)	-213*** (-253,-173)	-200*** (-241,-159)	-316*** (-486,-146)
Subgroup: number of visits <20 (N=383)					
Total outpatient costs	-29*** (-46,-12)	-36*** (-56,-16)	-45*** (-67,-22)	-18 (-36,1)	-231*** (-337,-125)
Reimbursed outpatient costs	1 (-8,10)	2 (-9,13)	0 (-12,12)	0 (-10,10)	-14 (-71,44)
Out-of-pocket outpatient costs	-30*** (-42,-18)	-38*** (-53,-24)	-44*** (-61,-28)	-18* (-31,-4)	-218*** (-293,-142)
Association between continuity of care measures and inpatient costs					
Subgroup: number of visits >=20					
Any inpatient cost, OR (95% CI) (N=1,023)	0.79*** (0.70,0.88)	0.75*** (0.65,0.85)	0.74*** (0.64,0.85)	0.79*** (0.71,0.89)	0.23*** (0.12,0.44)
Total conditional inpatient costs (N =361)	-562 (-1556,432)	-582 (-1603,438)	-548 (-1634,539)	-967 (-2068,134)	-5386* (-10556,-217)
Reimbursed conditional inpatient costs (N =358)	-67 (-731,597)	-71 (-753,610)	-50 (-774,673)	-349 (-1083,385)	-2798 (-6261,665)
Out-of-pocket conditional inpatient costs (N =361)	-470* (-924,-17)	-486* (-951,-21)	-479 (-974,16)	-598* (-1100,-96)	-2462* (-4828,-97)
Subgroup: number of visits <20					
Any inpatient cost, OR	0.79***	0.75***	0.74***	0.79***	0.23***

(95% CI) (N=383)	(0.70,0.88)	(0.65,0.85)	(0.64,0.85)	(0.71,0.89)	(0.12,0.44)
Total conditional inpatient costs (N=84)	-1831* (-3226,-436)	-2177** (-3755,-598)	-2484** (-4151,-817)	-1063 (-2598,472)	-5385 (-13613,2844)
Reimbursed conditional inpatient costs (N=84)	-779** (-1336,222)	-917** (-1547,286)	-1024** (-1691,-357)	-425 (-1041,192)	-1758 (-5077,1561)
Out-of-pocket conditional inpatient costs (N=84)	-1052* (-1975,-129)	-1260* (-2305,-216)	-1460* (-2564,-356)	-638 (-1644,368)	-3627 (-9004,1751)

*p<0.05, **p<0.01, ***p<0.001.

Ordinary least squares models adjusted for age, sex, village, medical insurance program, chronic diseases, number of total outpatient visits, number of total outpatient visits squared.

CI indicates confidence interval; COC, Bice-Boxerman Continuity of Care Index; coef, coefficient; HI, Herfindahl Index; PCP-UPC, Having a primary care provider as the usual provider of care; SECON, Sequential Continuity Index; UPC, Usual Provider of Care.
