# Supplementary material for: Continuity of Care and Healthcare Costs among Patients with Chronic Disease: Evidence from Primary Care Settings in China
Source: Int J Integr Care. 2022 Oct 12;22(4):4. doi: 10.5334/ijic.5994 (PMC9562970; doi:10.5334/ijic.5994)
Supplement: Additional file 8. — Table which presents the association between the first-year continuity of care and the second-year outpatient costs.docx. [file ijic-22-4-5994-s8.pdf]

Additional file 8. The association between the first-year continuity of care and the second-year outpatient costs among 1316 patients in Yuhuan City between September 2017 and August 2019

Primary predictors, coef (95% CI)	COC	HI	UPC	SECON	PCP-UPC
Total costs	-134*** (-203, -65)	-169*** (-249,-90)	-215*** (-307,-124)	-183*** (-256,-110)	-448* (-854,-43)
Reimbursed costs	-50* (-96,-5)	-67* (-119,-15)	-91** (-151,-30)	-81** (-129,-33)	-131 (-399,136)
Out-of-pocket costs	-84*** (-114,-53)	-103*** (-138,-67)	-124*** (-165,-84)	-102*** (-134,-69)	-317** (-499,-135)

*p<0.05, **p<0.01, ***p<0.001
Ordinary least squares models adjusted for age, sex, village, medical insurance program, chronic diseases, number of total outpatient visits, number of total outpatient visits squared.
CI indicates confidence interval; COC, Bice-Boxerman Continuity of Care Index; coef, coefficient; HI, Herfindahl Index; PCP-UPC, Having a primary care provider as the usual provider of care; SECON, Sequential Continuity Index; UPC, Usual Provider of Care.
