# Supplementary material for: Continuity of Care and Healthcare Costs among Patients with Chronic Disease: Evidence from Primary Care Settings in China
Source: Int J Integr Care. 2022 Oct 12;22(4):4. doi: 10.5334/ijic.5994 (PMC9562970; doi:10.5334/ijic.5994)
Supplement: Additional file 10. — Table which presents the full regression results of the hospitalization risk.docx. [file ijic-22-4-5994-s10.pdf]

Additional file 10. The full regression results of the hospitalization risk among 1406 patients in Yuhuan City between September 2017 and August 2019

	OR（95% CI）				
	COC	HI	UPC	SECON	PCP-UPC
Continuity of care measures	0.77*** (0.72,0.82)	0.74*** (0.69,0.79)	0.74*** (0.69,0.80)	0.79*** (0.74,0.85)	0.32*** (0.24,0.44)
Sex	1.00 (0.71,1.18)	1.00 (0.71,1.18)	1.00 (0.71,1.19)	1.00 (0.73,1.22)	1.00 (0.74,1.23)
Age	1.05*** (1.04,1.07)	1.06*** (1.04,1.07)	1.05*** (1.04,1.07)	1.05*** (1.04,1.07)	1.06*** (1.04,1.07)
Village/community					
1	1.00 (0.36,1.07)	1.00 (0.34,1.02)	1.00 (0.37,1.09)	1.00 (0.44,1.28)	2.00 (0.89,2.62)
2	0.49** (0.30,0.80)	0.46** (0.28,0.76)	0.49** (0.30,0.80)	0.57* (0.36,0.93)	1.00 (0.51,1.27)
3	0.39*** (0.23,0.66)	0.37*** (0.22,0.63)	0.40*** (0.24,0.67)	0.44** (0.26,0.74)	1.00 (0.61,1.72)
4	1.00 (0.42,1.47)	1.00 (0.39,1.38)	1.00 (0.43,1.50)	1.00 (0.52,1.80)	2.41** (1.28,4.53)
5	0.53* (0.32,0.86)	0.53* (0.32,0.87)	0.52** (0.32,0.86)	0.54* (0.33,0.89)	0.57* (0.35,0.92)
6	1.00 (0.38,1.18)	1.00 (0.36,1.14)	1.00 (0.39,1.21)	1.00 (0.46,1.40)	1.89* (1.07,3.34)
7	1.00 (0.39,1.06)	1.00 (0.38,1.04)	1.00 (0.39,1.07)	1.00 (0.42,1.14)	1.00 (0.55,1.48)
Having Hypertension only	0.73* (0.54,0.98)	0.74* (0.55,0.99)	1.00 (0.56,1.00)	1.00 (0.59,1.06)	1.00 (0.56,1.01)
Having diabetes only	1.00 (0.75,2.02)	1.00 (0.75,2.04)	1.00 (0.75,2.03)	1.00 (0.78,2.09)	1.00 (0.78,2.08)
Resident Basic Medical Insurance	2.01** (1.21,3.32)	2.01** (1.21,3.32)	1.96** (1.19,3.25)	1.97** (1.19,3.25)	2.00 (0.97,2.54)
Number of outpatient encounters	1.03*** (1.02,1.04)	1.03*** (1.02,1.03)	1.03*** (1.02,1.04)	1.03*** (1.02,1.04)	1.03*** (1.02,1.03)

Number of outpatient encounters squared	1.00*** (1.00,1.00)	1.00*** (1.00,1.00)	1.00*** (1.00,1.00)	1.00*** (1.00,1.00)	1.00*** (1.00,1.00)
Constant	0.03*** (0.01,0.08)	0.04*** (0.01,0.12)	0.05*** (0.02,0.17)	0.02*** (0.01,0.07)	0.01*** (0.00,0.02)

CI indicates confidence interval; COC, Bice-Boxerman Continuity of Care Index; HI, Herfindahl Index; OR, odds ratio; PCP-UPC, Having a primary care provider as the usual provider of care; SECON, Sequential Continuity Index; UPC, Usual Provider of Care.
