# Supplementary material for: Continuity of Care and Healthcare Costs among Patients with Chronic Disease: Evidence from Primary Care Settings in China
Source: Int J Integr Care. 2022 Oct 12;22(4):4. doi: 10.5334/ijic.5994 (PMC9562970; doi:10.5334/ijic.5994)
Supplement: Additional file 11. — Table which presents the full regression results of the conditional inpatient costs.docx. [file ijic-22-4-5994-s11.pdf]

Additional file 11. The full regression results of the conditional inpatient costs among 1406 patients in Yuhuan City between September 2017 and August 2019

**Total**	coef（95% CI）				
	COC	HI	UPC	SECON	PCP-UPC
Continuity of care measures	-773 (-1563,18)	-824 (-1657,9)	-830 (-1714,53)	-889* (-1758,-21)	-4887* (-9146,-628)
Sex	-882 (-4011,2247)	-883 (-4012,2246)	-797 (-3924,2329)	-761 (-3884,2363)	-940 (-4066,2186)
Age	73 (-70,217)	74 (-69,217)	73 (-70,216)	82 (-62,226)	70 (-73,212)
Village/community					
1	1110 (-5450,7670)	1057 (-5509,7622)	1199 (-5356,7753)	1294 (-5239,7826)	3873 (-2835,10581)
2	6056* (410,11701)	5995* (339,11651)	6183* (555,11812)	6283* (698,11867)	6852* (1354,12350)
3	-249 (-6667,6170)	-297 (-6721,6128)	-102 (-6500,6296)	-274 (-6679,6131)	3162 (-3275,9600)
4	2510 (-4772,9792)	2508 (-4770,9787)	2649 (-4618,9917)	2751 (-4479,9982)	6930 (-717,14577)
5	1325 (-4431,7080)	1330 (-4424,7085)	1358 (-4398,7114)	1659 (-4092,7411)	1375 (-4369,7120)
6	2151 (-4475,8778)	2070 (-4571,8710)	2204 (-4425,8833)	2398 (-4166,8963)	5451 (-1220,12122)
7	3118 (-2727,8963)	3096 (-2750,8941)	3190 (-2653,9034)	3234 (-2603,9071)	3300 (-2527,9128)
Having Hypertension only	-3526* (-6988,-64)	-3525* (-6987,-64)	-3460.23* (-6920,-1)	-3231 (-6685,222)	-3404 (-6854,46)
Having diabetes only	-4474 (-10639,1691)	-4489 (-10653,1675)	-4508 (-10674,1658)	-4147 (-10327,2032)	-4490 (-10644,1664)
Resident Basic Medical Insurance	-6570* (-12319,-822)	-6591* (-12334,-848)	-6747* (-12474,-1019)	-6468* (-12224,-712)	-7208* (-12873,-1542)
Number of outpatient encounters	-26 (-117,65)	-32 (-123,60)	-29 (-120,63)	-28 (-119,63)	-41 (-133,51)
Number of outpatient	0 (0,0)	0 (0,0)	0 (0,0)	0 (0,0)	0 (0,0)

encounters squared					
Constant	17807* (3786,31828)	18443* (4277,32609)	19374** (4846,33901)	18412* (4291,32534)	15073* (1422,28724)

**Reimbursed**	coef（95% CI)				
	COC	HI	UPC	SECON	PCP-UPC
Continuity of care measures	-168 (-676,341)	-187 (-722,349)	-168 (-735,399)	-277 (-834,280)	-2286 (-5026,454)
Sex	-603 (-2611,1406)	-605 (-2613,1403)	-582 (-2588,1424)	-581 (-2584,1423)	-680 (-2684,1324)
Age	77 (-15,169)	77 (-14,169)	77 (-15,169)	81 (-11,174)	79 (-12,171)
Village/community					
1	567 (-3630,4764)	547 (-3653,4747)	597 (-3596,4789)	545 (-3634,4723)	1653 (-2637,5944)
2	3953* (341,7566)	3927* (309,7546)	3998* (398,7598)	3902* (330,7474)	4005* (491,7518)
3	-359 (-4465,3747)	-384 (-4495,3726)	-308 (-4400,3784)	-510 (-4607,3587)	861 (-3256,4978)
4	1093 (-3565,5752)	1081 (-3576,5738)	1140 (-3508,5788)	1045 (-3580,5670)	2839 (-2056,7734)
5	1266 (-2460,4992)	1264 (-2462,4990)	1278 (-2448,5004)	1347 (-2375,5068)	1223 (-2492,4938)
6	511 (-3729,4750)	479 (-3769,4727)	541 (-3699,4781)	455 (-3744,4654)	1709 (-2558,5976)
7	1021 (-2718,4760)	1013 (-2727,4752)	1040 (-2697,4778)	1027 (-2706,4759)	1025 (-2699,4749)
Having Hypertension only	-2751* (-4973,-529)	-2753* (-4974,-531)	-2736* (-4957,-516)	-2681* (-4898,-464)	-2739* (-4952,-526)
Having diabetes only	-2610 (-6598,1377)	-2613 (-6601,1374)	-2622 (-6610,1366)	-2490 (-6486,1506)	-2557 (-6535,1420)
Resident Basic Medical Insurance	-8309*** (-11999,-4619)	-8305*** (-11992,-4619)	-8360*** (-12035,-4685)	-8191*** (-11884,-4499)	-8367*** (-11999,-4735)
Number of outpatient encounters	-38 (-97,20)	-40 (-98,19)	-39 (-97,20)	-39 (-98,19)	-45 (-105,14)

Number of outpatient encounters squared	0 (0,0)	0 (0,0)	0 (0,0)	0 (0,0)	0 (0,0)
Constant	11250* (2259,20241)	11424* (2338,20510)	11523* (2206,20841)	11724* (2673,20774)	10729* (1990,19468)

**Out-of-pocket**	coef（95% CI)				
	COC	HI	UPC	SECON	PCP-UPC
Continuity of care measures	-589** (-971,-206)	-620** (-1023,-217)	-648** (-1076,-221)	-598** (-1019,-177)	-2515* (-4587,-443)
Sex	-274 (-1788,1240)	-273 (-1787,1241)	-211 (-1724,1301)	-176 (-1690,1337)	-260 (-1780,1261)
Age	-3 (-72,66)	-3 (-72,67)	-3 (-72,67)	1 (-69,71)	-9 (-78,60)
Village/community					
1	551 (-2623,3724)	518 (-2658,3695)	605 (-2566,3776)	749 (-2418,3916)	2179 (-1085,5442)
2	2129 (-602,4861)	2095 (-641,4832)	2205 (-518,4928)	2397 (-310,5104)	2857* (183,5532)
3	138 (-2967,3243)	116 (-2993,3224)	224 (-2871,3320)	256 (-2848,3361)	2267 (-865,5399)
4	1430 (-2093,4953)	1441 (-2081,4963)	1515 (-2001,5031)	1709 (-1796,5215)	4028* (307,7748)
5	248 (-2536,3033)	254 (-2530,3038)	271 (-2514,3055)	490 (-2298,3278)	321 (-2473,3116)
6	1661 (-1545,4866)	1612 (-1601,4825)	1676 (-1531,4883)	1951 (-1231,5134)	3704* (458,6949)
7	2101 (-727,4929)	2087 (-741,4916)	2152 (-675,4979)	2208 (-621,5038)	2276 (-560,5111)
Having Hypertension only	-819 (-2494,856)	-816 (-2491,858)	-772 (-2445,902)	-601 (-2275,1074)	-700 (-2379,978)
Having diabetes only	-1841 (-4824,1141)	-1854 (-4837,1128)	-1865 (-4848,1118)	-1637 (-4633,1358)	-1893 (-4887,1101)
Resident Basic Medical	1745 (-1036,4526)	1721 (-1058,45	1625 (-1146,43	1730 (-1060,45	1185 (-1571,3942)

Insurance		00)	96)	20)	
Number of outpatient encounters	12<br>(-32,56)	8<br>(-36,52)	10<br>(-34,54)	11<br>(-33,55)	5<br>(-40,50)
Number of outpatient encounters squared	0<br>(0,0)	0<br>(0,0)	0<br>(0,0)	0<br>(0,0)	0<br>(0,0)
Constant	6428<br>(-355,13211)	6879*<br>(25,13734<br>)	7708*<br>(680,1473<br>6)	6563<br>(-283,134<br>08)	4268<br>(-2373,10910<br>)

CI indicates confidence interval; COC, Bice-Boxerman Continuity of Care Index; Coef, coefficient; HI, Herfindahl Index; PCP-UPC, Having a primary care provider as the usual provider of care; SECON, Sequential Continuity Index; UPC, Usual Provider of Care.
