# Supplementary material for: Continuity of Care and Healthcare Costs among Patients with Chronic Disease: Evidence from Primary Care Settings in China
Source: Int J Integr Care. 2022 Oct 12;22(4):4. doi: 10.5334/ijic.5994 (PMC9562970; doi:10.5334/ijic.5994)
Supplement: Additional file 12. — Figure which demonstrates reduced hospitalization risk when setting continuity of care measures to 1 compared to status quo.docx. [file ijic-22-4-5994-s12.pdf]

Additional file 12. Reduced hospitalization risk when setting continuity of care to 1 compared to status quo among 1406 patients in Yuhuan City between September 2017 and August 2019.

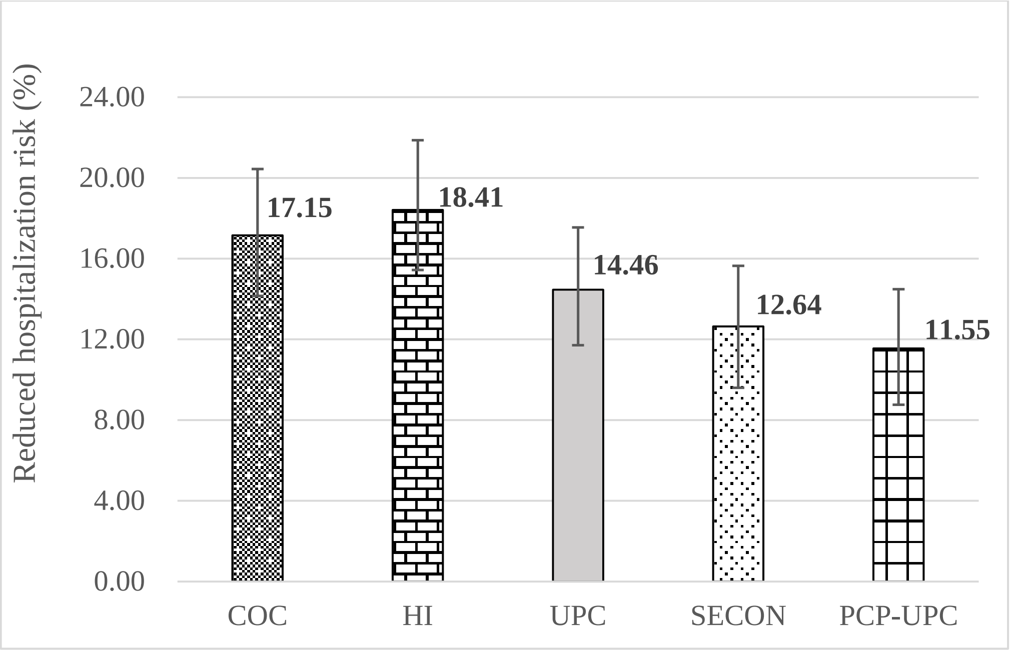

COC indicates Bice-Boxerman Continuity of Care Index; HI, Herfindahl Index; PCP-UPC, Having a primary care provider as the usual provider of care; SECON, Sequential Continuity Index; UPC, Usual Provider of Care.
