# Supplementary material for: Continuity of Care and Healthcare Costs among Patients with Chronic Disease: Evidence from Primary Care Settings in China
Source: Int J Integr Care. 2022 Oct 12;22(4):4. doi: 10.5334/ijic.5994 (PMC9562970; doi:10.5334/ijic.5994)
Supplement: Additional file 13. — Table which presents the association between the first-year continuity of care and the second-year inpatient costs.docx. [file ijic-22-4-5994-s13.pdf]

Additional file 13. The association between the first-year continuity of care and the second-year inpatient costs among 1316 patients in Yuhuan City between September 2017 and August 2019

Primary predictors, coef (95% CI)	COC	HI	UPC	SECON	PCP-UPC
Any cost, OR (95% CI)	0.93* (0.88,0.98)	0.91** (0.85,0.98)	0.91* (0.85,0.99)	0.91** (0.86,0.96)	0.87 (0.63,1.22)
Total conditional costs (n=267)	-961 (-2323,400)	-980 (-2534,574)	-1290 (-3051,470)	-995 (-2388,398)	-1521 (-9392,6351)
Reimbursed conditional costs (n=264)	-428 (-1145,288)	-498 (-1316,319)	-583 (-1509,343)	-454 (-1188,280)	-2067 (-6209,2075)
Out-of-pocket conditional costs (n=267)	-524 (-1286,238)	-471 (-1342,399)	-697 (-1683,289)	-533 (-1314,247)	601 (-3806,5008)

*p<0.05, **p<0.01, ***p<0.001

Ordinary least squares models adjusted for age, sex, village, medical insurance program, chronic diseases, number of total outpatient visits, number of total outpatient visits squared.

CI indicates confidence interval; COC, Bice-Boxerman Continuity of Care Index; coef, coefficient; HI, Herfindahl Index; OR, odds ratio; PCP-UPC, Having a primary care provider as the usual provider of care; SECON, Sequential Continuity Index; UPC, Usual Provider of Care.
